# Genome sequences of four colistin-resistant ESKAPE bacterial strains isolated from patients within the same hospital

**DOI:** 10.1128/mra.00874-23

**Published:** 2023-12-19

**Authors:** Merve Nur Tunç, Virgile Guéneau, Valentin Loux, Rosa del Campo, Rut Carballido Lopez, Romain Briandet

**Affiliations:** 1 Université Paris-Saclay, INRAE, AgroParisTech, Micalis Institute, Jouy-en-Josas, France; 2 Lallemand SAS, Blagnac, France; 3 Université Paris-Saclay, INRAE, MaIAGE, Jouy-en-Josas, France; 4 INRAE, BioinfOmics, MIGALE bioinformatics facility, Université Paris-Saclay, Jouy-en-Josas, France; 5 Servicio de Microbiología, Hospital Universitario Ramón y Cajal and Instituto Ramón y Cajal de Investigación Sanitaria, Madrid, Spain; Loyola University Chicago, Chicago, Illinois, USA

**Keywords:** *Pseudomonas aeruginosa*, *Klebsiella pneumoniae*, colistin-resistance, milti-drug resistant, ESKAPE

## Abstract

The genomes of four clinical Gram-negative ESKAPE bacterial strains highly resistant to the last-resort antibiotic colistin were sequenced and analyzed. The strains were found to carry multidrug-resistant genes besides colistin-resistant genes.

## ANNOUNCEMENT

Colistin (polymyxin E) is a last-resort antibiotic used against multidrug-resistant (MDR) Gram-negative bacteria. However, its efficacy is increasingly compromised by the emergence of colistin-resistant strains. Here, we sequenced the genomes of four colistin-resistant strains isolated from unrelated patients suspected of infection at the Microbiology Department of the Ramón y Cajal University Hospital of Madrid, Spain, with the approval from The Drug Research Ethics Committee of the hospital (CEIm, identification number 2017-163/17). The strains belong to two different species: two are of *Pseudomonas aeruginosa* and two of *Klebsiella pneumoniae*. The *P. aeruginosa* strains were isolated from the sputum of cystic fibrosis patients and *K. pneumoniae* strains from a patient with a urinary tract infection. Bacterial samples were grown on McConkey growth medium (Difco) at 37°C and identified by MALDI-TOF MS (Burker).

The minimum inhibitory concentration (MIC) for colistin was determined according to the EUCAST procedure, using the microdilution method with the cationic-adjusted Mueller–Hinton broth medium (Difco). The MIC values of all strains were well above the species breakpoint (2 mg/L for *K. pneumoniae* and 4 mg/L for *P. aeruginosa*), classifying them as colistin-resistant (Table 1).

**TABLE 1:** Genome assembly details and statistics of *P. aeruginosa* and *K. pneumoniae* isolated from patients within the same hospital

	PAMNT027	PAMNT030	PAMNT028	PAMNT034
Species	*P. aeruginosa*	*P. aeruginosa*	*K. pneumoniae*	*K. pneumoniae*
Genome size (bp)	6,237,612	6,227,686	5,320,190	5,599,993
The total read coverage (x)	141.0	197.7	140.9	129.2
No. of contigs	55	55	46	84
GC content (%)	66.54	66.53	57.51	57.17
No. of raw read pairs	5.823.045	8.152.234	4.966.094	4.790.919
*N*_50_ value (bp)	703,869	693,914	444,847	222,209
No. of CDS	5,746	5,735	5,095	5,500
No. of rRNAs	3	3	4	4
No. of tRNAs	57	57	74	77
Antimicrobial-resistant genes annotated by Staramr	*aph3′-IIb, blaOXA-486, blaPAO, catB7, fosA*	*aph3′-IIb, blaOXA-486, blaPAO, catB7, fosA*	*aac3-IId, aac6′-Ib-cr, aadA16, ARR-3, blaSHV-2, dfrA27, fosA16, OqxA, OqxB, qacE, qnrB6, sul1, tetD*	*aac3-IIa, aac6′-Ib-cr, aadA2, blaCTX-M-15, blaOXA-1, blaOXA-48, blaSHV-182, catA1, catB3, dfrA12, fosA, OqxA, OqxB, qacE, qnrB1, sul1*
Colistin resistance/tolerance related genes	*mexA, mexB, mexR, oprM, emrA, emrB, arnBCADTEF, kdsD, araG, eptA, eptC, phoQ, phoP*	*mexA,mexB, mexR, oprM, emrA, emrB, arnBCADTEF, kdsD, araG, eptA, eptC, phoQ, phoP*	*mexA,mexB, oprM, emrA, emrB, arnBCADTEF, kdsD, araA, araC, araE, araF, araG, araH, gutQ, eptA, eptB, opgE, phoQ, phoP*	*mexA,mexB, oprM, emrA, emrB, arnBCADTEF, kdsD, araA, araC, araE, araF, araG, araH, gutQ, eptA, eptB, opgE, phoQ, phoP*
MIC for colistin	32	16	256	256
Plasmid finder	–	–	lncR	lncFIB(K), lncR
Accession no.	JASERN000000000	JASERO000000000	JASERQ000000000	JASERR000000000

For genome sequencing, strains were retrieved from −70°C glycerol stocks and cultured overnight in Lysogenic Broth medium at 37°C with agitation. Genomic DNA was extracted using the GenElute Bacterial Genomic DNA Kits (Sigma-Aldrich), quantified using the Qubit dsDNA Kit (Thermo Fisher Scientific) and sent to Eurofins Genomics Europe Sequencing (Germany) for sequencing. DNA-seq libraries were prepared according to Eurofins Illumina’s protocol and sequenced on NovaSeq 6000 with 2 × 150 bp paired-end read mode and output of approximately 5 million paired-end reads per sample. Reads quality was checked by FastQC (version:0.11.9) (1).

The reads were analyzed using the Galaxy software (https://galaxy.migale.inrae.fr/) (2). For each tool, default parameters were used except where otherwise stated. *De novo* assembly was performed using Unicycler (Galaxy version:0.4.8.0) (3) and quality control using Quast (Galaxy version:5.0.2+galaxy4) (4 – 7). Genome annotation was completed using the NCBI Prokaryotic Genome Annotation Pipeline (PGAP version:6.5) (8 – 10). Total genome size, percent genome coverage, number of contigs, raw read pairs, CDS, rRNAs, tRNAs, GC content, N50 value, and the identified plasmids are listed in Table 1.

In addition to antimicrobial resistant genes annotated by Staramr (Galaxy version:0.8.0+galaxy0) (11), genes known to be associated with colistin tolerance/resistance mechanisms in the annotated genome sequences are listed in Table 1. Colistin is a cationic polypeptide that interacts with phosphate groups of the lipopolysaccharide (LPS) in the outer membrane. The mechanisms of resistance mainly involve modifications of the LPS target (12) or export of the antibiotic by multidrug efflux (Mex) systems (13). The colistin resistance-related genes *arnBCADTEF, kdsD, eptA, phoPQ* which are involved in the addition of either 4-amino-4-deoxy-l-arabinose (l-Ara4N) or phosphoethanolamine (pEtN) to the LPS to reduce its negative charge and thus its affinity for colistin (12, 14, 15) are present in all four genomes. The regulatory genes *mexAB, emrAB*, encoding components of the MDR efflux pumps Mex and MFS (13, 16, 17) are also present in all four genomes.

Two *Pseudomonas aeruginosa* and two *Klebsiella pneumoniae* colistin-resistant clinical strains were isolated from unrelated patients under suspicion of infection at the Microbiology Department of the Ramón y Cajal University Hospital of Madrid, Spain. *P. aeruginosa* strains were isolated from sputum of cystic fibrosis patients and *K. pneumoniae* strains were obtained from a patient with urinary tract infection. The four isolates were sequenced by INVIEW Illumina sequencing with 2 × 150 bp paired-end read mode and output of approximately 5 million read pairs. DNAseq libraries were prepared according to Illumina’s protocol.

## Data Availability

This Whole Genome Shotgun project has been deposited at DDBJ/ENA/GenBank under the accession numbers JASERN000000000, JASERQ000000000, JASERO000000000, JASERR000000000 for strains PAMNT027, PAMNT028, PAMNT030, and PAMNT034 respectively and the draft genome assembly and annotation can be found in NCBI under BioProject number PRJNA966919 and SRA numbers, SRR25684271, SRR25684269, SRR25684270, SRR25684268 for PAMNT027, PAMNT028, PAMNT030, and PAMNT034 respectively. The versions described in this paper are versions JASERN010000000, JASERQ010000000, JASERO010000000 and JASERR010000000 for PAMNT027, PAMNT028, PAMNT030, and PAMNT034 respectively.
